# Cholestatic Jaundice as the Initial Manifestation of Classical Hodgkin Lymphoma: A Diagnostic Challenge

**DOI:** 10.7759/cureus.97264

**Published:** 2025-11-19

**Authors:** Idan Grossmann, Petro Vavrukh, Jasminka Balderacchi, Sidra Ahsan, Harshavardhan Sanekommu

**Affiliations:** 1 Internal Medicine, Hackensack Meridian Health Jersey Shore University Medical Center, Neptune, USA; 2 Pathology, Cooperman Barnabas Medical Center, Livingston, USA; 3 Pathology, Monmouth Medical Center, Long Branch, USA; 4 Gastroenterology and Hepatology, Hackensack Meridian Health Jersey Shore University Medical Center, Neptune, USA

**Keywords:** cholestatic jaundice, diagnostic challenge, liver biopsy, liver infiltration, liver-involved hodgkin lymphoma

## Abstract

Hodgkin lymphoma (HL) is a cancer of the lymphatic system that typically presents with systemic B symptoms and lymphadenopathy, while hepatic involvement is rare, and initial presentation with liver dysfunction is uncommon. We report a case of a 47-year-old patient who presented with progressive jaundice and generalized weakness. His clinical course and laboratory findings suggested multiple possible causes, and due to diagnostic uncertainty, a liver biopsy was performed, revealing Hodgkin and Reed-Sternberg cells consistent with classical HL. This case highlights a rare initial hepatic presentation of HL manifested as cholestatic jaundice and underscores the importance of considering infiltrative malignancies in the differential diagnosis of unexplained liver dysfunction, as well as the critical role of liver biopsy when noninvasive evaluations fail to provide a conclusive diagnosis.

## Introduction

Hodgkin lymphoma (HL) is a B-cell-derived malignancy that accounts for approximately 10-15% of all lymphoma diagnoses. The disease is characterized by the presence of distinct mononuclear Hodgkin cells and bi- and multinucleated Reed-Sternberg cells [1]. The etiology of HL is multifactorial and not well understood. However, several risk factors have been established, including Epstein-Barr virus (EBV) infection, immunodeficiency, autoimmune conditions, and genetic susceptibility [2]. Liver involvement as the initial presentation of HL is extremely rare, occurring in about 1-2% of cases, and poses significant diagnostic challenges due to its ability to mimic other causes of liver dysfunction. This atypical presentation often manifests as progressive jaundice, generalized weakness, and significant weight loss, which can obscure the underlying diagnosis of Hodgkin lymphoma [3]. The majority of HL cases present with painless lymphadenopathy and B symptoms (fever, night sweats, weight loss), with extranodal involvement, seen in approximately 15% of cases. Liver involvement is reported in about 5-8% of newly diagnosed HL cases, but it is almost always a marker of advanced disease [4]. Therefore, while 5-8% of patients have liver infiltration at diagnosis, its presentation as the initial manifestation of HL is far less common, occurring in only 1-2% of cases. This atypical presentation poses significant diagnostic challenges as clinical, laboratory, and imaging findings are frequently non-specific and may be mistaken for infections, autoimmune, or other malignant liver diseases [5,6]. This case report highlights the diagnostic challenges of atypical liver involvement in HL and emphasizes the importance of considering infiltrative malignancies in the differential diagnosis of unexplained liver dysfunction.

## Case presentation

A 47-year-old man with a past medical history of type 2 diabetes mellitus and hyperlipidemia presented with progressively worsening jaundice and generalized weakness that began abruptly three weeks before admission. The patient reported nausea, vomiting, anorexia, and an unintentional weight loss of approximately 50 pounds over a month. He denied alcohol consumption, illicit drug use, needle exposure, unsafe sexual practices, recent travel, autoimmune disease, inflammatory bowel disease, or a history of malignancy. On admission, vital signs were notable for a fever of 101.2°F, blood pressure of 98/65 mmHg, and oxygen saturation of 96% on room air. Physical examination showed diffuse jaundice and bilateral lower extremity edema. Laboratory studies showed pancytopenia with a white blood cell (WBC) count of 4.4 ×10³/µL, hemoglobin at 9.4 g/dL (baseline 13.8 g/dL), and platelets at 109 ×10³/µL. Liver function tests revealed marked direct hyperbilirubinemia (total 13.3 mg/dL, direct 10 mg/dL), mildly elevated transaminases (aspartate aminotransferase (AST) 135 U/L, alanine transaminase (ALT) 164 U/L), elevated alkaline phosphatase (232 U/L) and gamma-glutamyl transferase (GGT) (100 U/L), and hypoalbuminemia (1.4 g/dL). Additional abnormalities included elevated lactate dehydrogenase (LDH) (327 U/L), ferritin >16,500 ng/mL, ceruloplasmin 38 mg/dL, and alpha-1-antitrypsin 348 mg/dL. (Table 1). Viral serologies were negative for hepatitis B, hepatitis C, and HIV. EBV serologies showed elevated IgG at 251 with normal IgM. Autoimmune testing, including antinuclear antibody (ANA), antineutrophil cytoplasmic antibodies (ANCA), anti-smooth muscle antibody, and anti-mitochondrial antibody, was negative. Tick-borne serologies for *Ehrlichia*, babesia, and Lyme disease were also negative (Table 2).

**Table 1:** Initial laboratory findings on admission WBC: white blood cell count; AST: aspartate aminotransferase; ALT: alanine aminotransferase; GGT: gamma-glutamyl transferase; LDH: lactate dehydrogenase

Parameter	Result	Reference Range
WBC	4.4 ×10³/µL	4.0–11.0 ×10³/µL
Hemoglobin	9.4 g/dL	13.5–17.5 g/dL
Platelets	109 ×10³/µL	150–450 ×10³/µL
Bandemia	15.8%	<10%
Neutrophils	75.4%	40–70%
Total bilirubin	13.3 mg/dL	0.2–1.2 mg/dL
Direct bilirubin	10.0 mg/dL	<0.3 mg/dL
AST	135 U/L	10–40 U/L
ALT	164 U/L	7–56 U/L
Alkaline phosphatase	232 U/L	44–147 U/L
GGT	100 U/L	0–65 U/L
Albumin	1.4 g/dL	3.5–5.0 g/dL
LDH	327 U/L	140–280 U/L
Ferritin	>16,500 ng/mL	30–400 ng/mL
Ceruloplasmin	38 mg/dL	20–60 mg/dL
Alpha-1-antitrypsin	348 mg/dL	85–220 mg/dL

**Table 2:** Viral, autoimmune, and tick-borne serologies HIV: human immunodeficiency virus; EBV: Epstein-Barr virus; ANA: anti-nuclear antibody; ANCA: anti-neutrophil cytoplasmic antibody

Test	Result
Hepatitis B surface antigen	Negative
Hepatitis C antibody	Negative
HIV	Negative
EBV IgG	Positive
EBV IgM	Normal
ANA	Negative
ANCA	Negative
Anti–smooth muscle antibody	Negative
Anti-mitochondrial antibody	Negative
Ehrlichia	Negative
Babesia	Negative
Lyme disease	Negative

**Figure 1:**
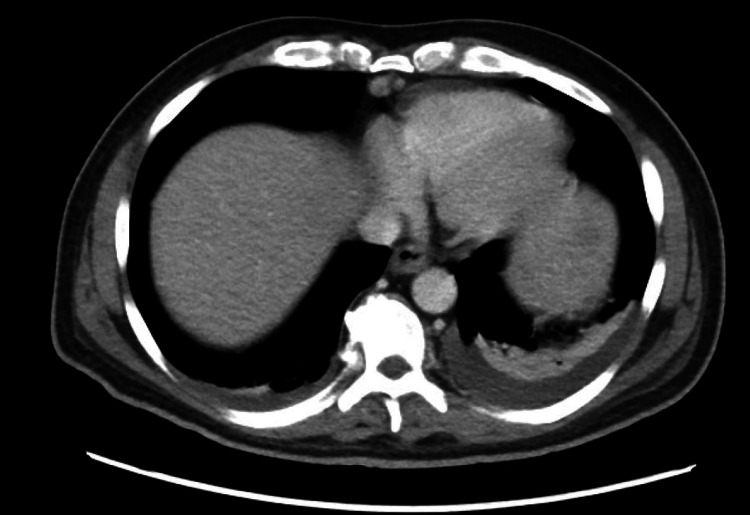
CT scan of abdomen and pelvis demonstrating small bilateral pleural effusions, more pronounced on the left side

**Figure 2:**
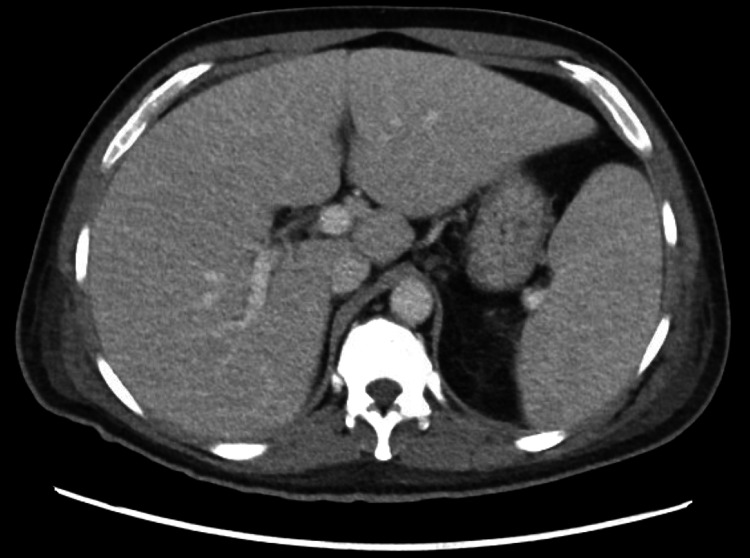
CT abdomen and pelvis demonstrating enlarged spleen (splenomegaly)

Imaging included a chest X-ray that was unremarkable. A CT of the abdomen and pelvis demonstrated small bilateral pleural effusions (Figure 1), splenomegaly (Figure 2), and a small amount of pericholecystic fluid. A right upper quadrant ultrasound revealed gallbladder wall thickening, and a hepatobiliary iminodiacetic acid (HIDA) scan suggested possible chronic cholecystitis. Magnetic resonance cholangiopancreatography (MRCP) showed no evidence of choledocholithiasis or biliary obstruction.

The patient was empirically started on piperacillin-tazobactam. Given persistent direct hyperbilirubinemia, hypoalbuminemia, markedly elevated ferritin, and the absence of a viral or obstructive etiology, an infiltrative hepatic process was suspected. Prior to pursuing liver biopsy, coagulation studies were obtained and demonstrated a prolonged prothrombin time (PT) of 21.2 seconds and an international normalized ratio (INR) of 1.81, consistent with impaired hepatic synthetic function. These abnormalities were corrected before biopsy to ensure procedural safety. A liver biopsy was performed for further evaluation.

**Figure 3:**
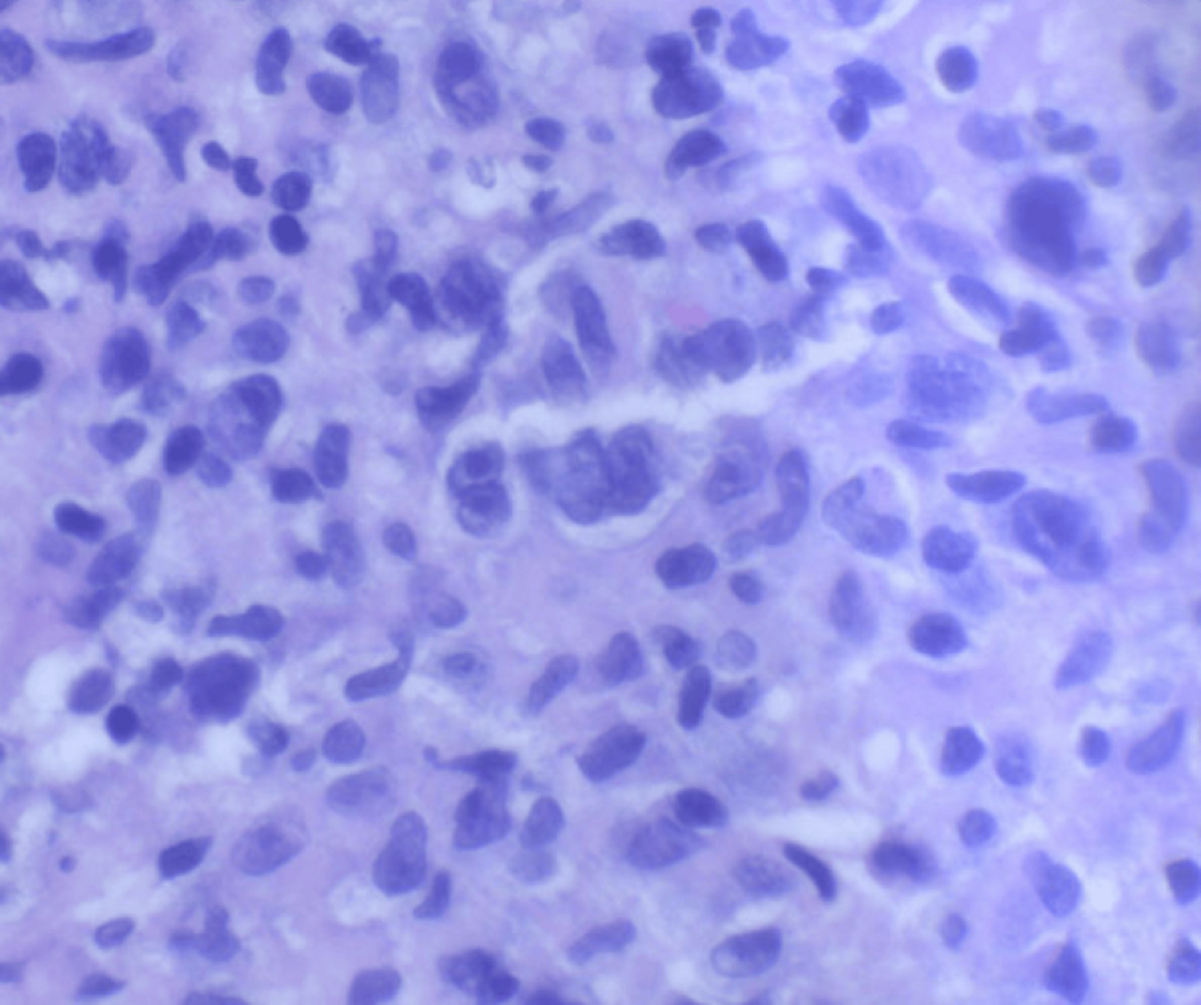
The owl's eye appearance of this bilobed cell with large inclusion-like nucleoli is characteristic of a classic Reed-Sternberg cell (hematoxylin and eosin stain, 400×).

**Figure 4:**
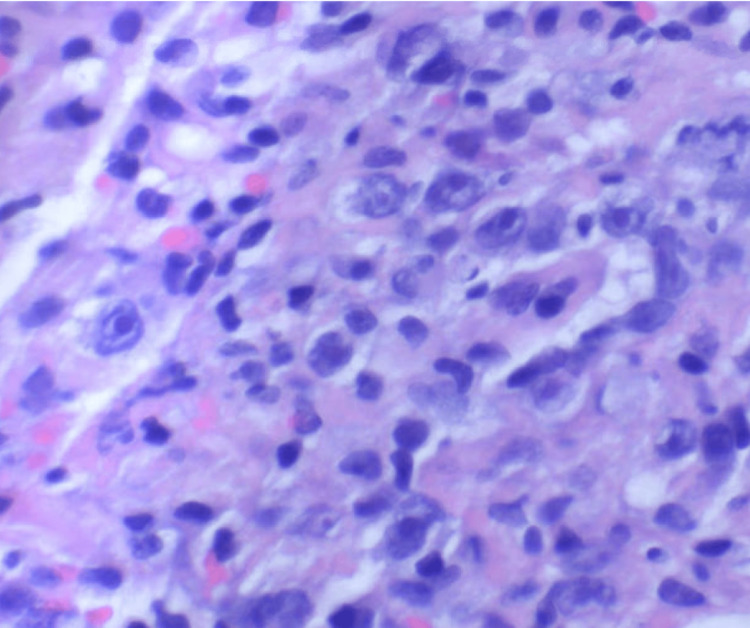
Mononuclear Reed-Sternberg cell variants are predominant in the liver biopsy specimen (hematoxylin and eosin stain, 400×).

**Figure 5:**
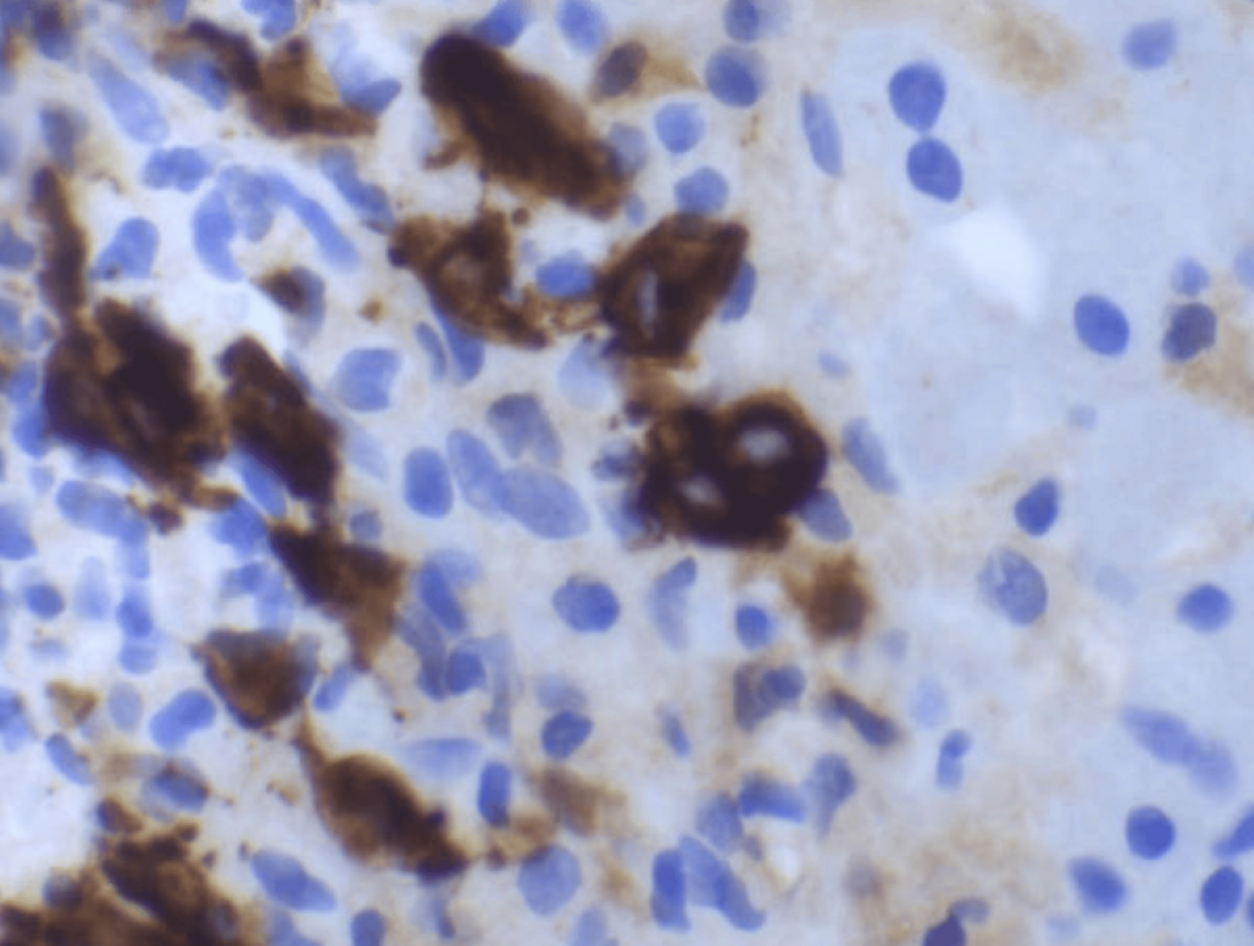
Reed-Sternberg cells demonstrate cytoplasmic immunoreactivity for CD30. Classic Reed-Sternberg cells and other Hodgkin cell variants all demonstrate a similar immunohistochemical staining profile (CD30, 400x).

**Figure 6:**
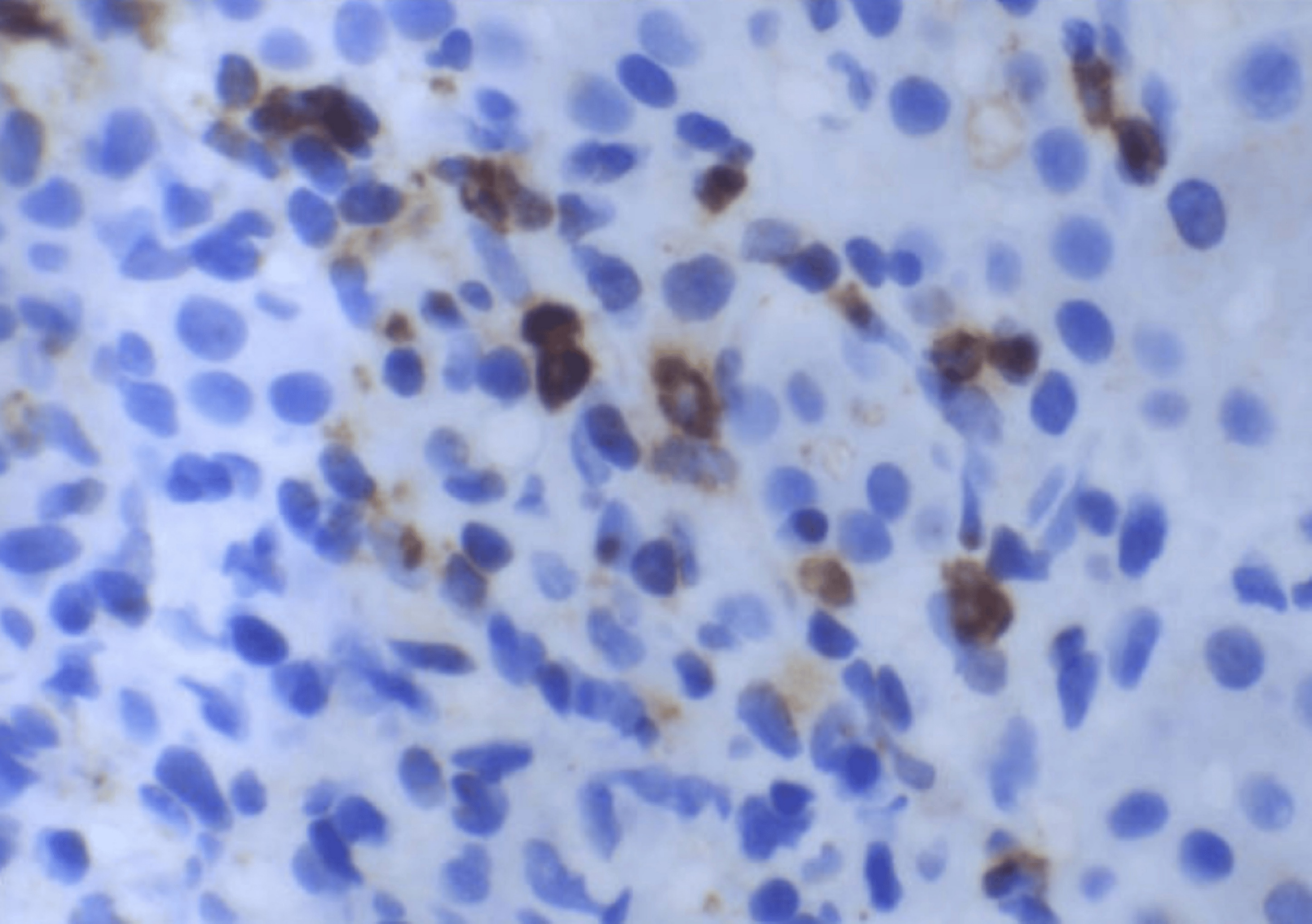
Reed-Sternberg cells demonstrate cytoplasmic immunoreactivity for CD15. Classic Reed-Sternberg cells and other Hodgkin cell variants all demonstrate a similar immunohistochemical staining profile (CD15, 400x).

**Figure 7:**
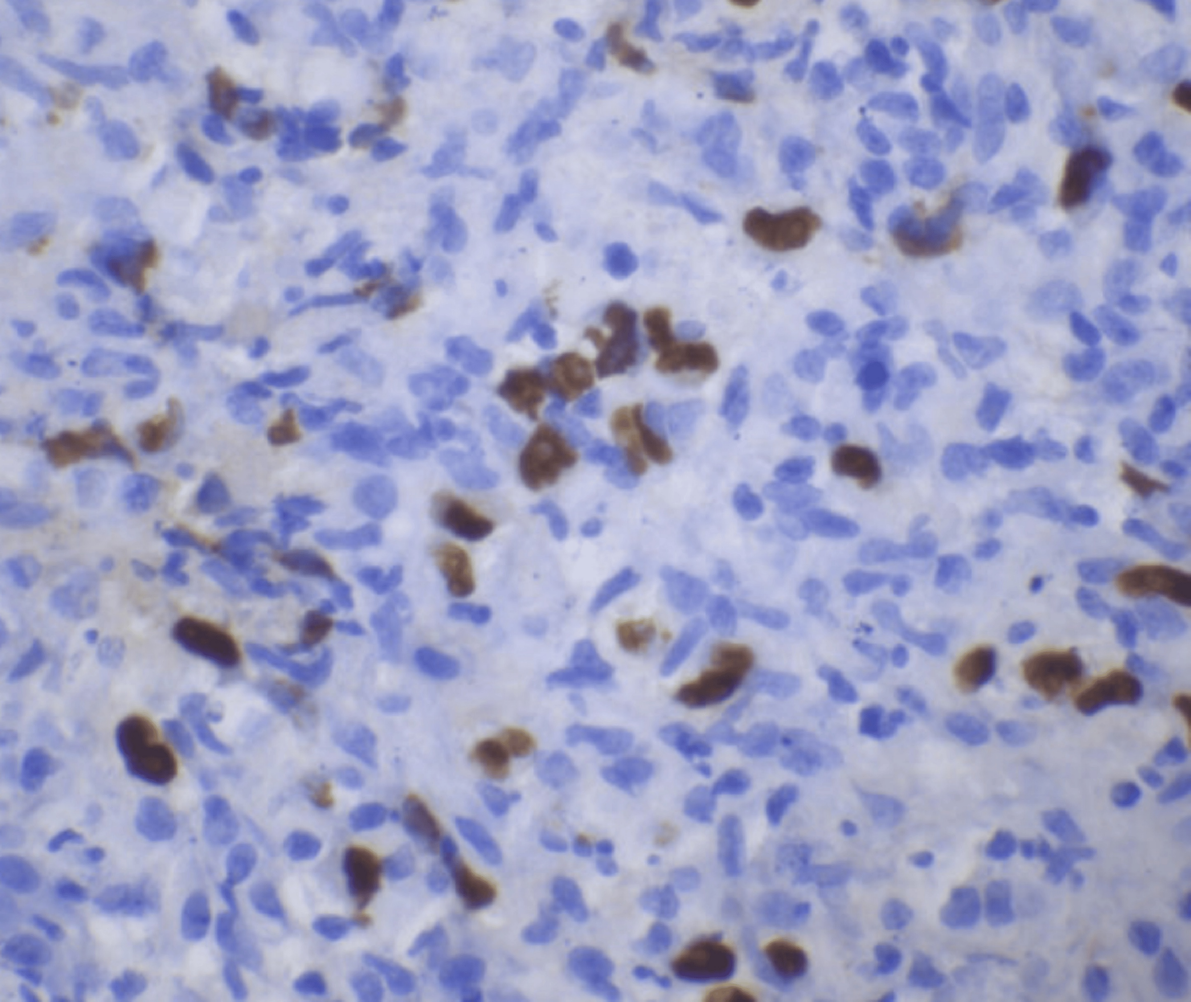
Reed-Sternberg cells demonstrate immunoreactivity for EBER. Classic Reed-Sternberg cells and other Hodgkin cell variants all demonstrate a similar immunohistochemical staining profile (EBER, 400x). EBER: Epstein-Barr virus-encoded RNAs

Liver core biopsy showed portal-centered polymorphous lymphohistiocytic aggregates containing scattered large mononuclear and bi/multinucleated Hodgkin/Reed-Sternberg (HRS)-type cells (Figures 3-7). The atypical cells expressed CD30 and CD15 with weak PAX5 and MUM1 positivity, are EBV-encoded small RNAs (EBER)-positive, and showed weak BCL6/BCL2; they lack CD45, CD20, and CD3. The background was rich in CD3+CD5+ small T cells, and Ki-67 labeling was highest in the HRS cells. Findings were diagnostic of classic Hodgkin lymphoma, mixed cellularity type, involving the liver. The patient was discharged with plans for outpatient oncology evaluation and initiation of chemotherapy.

## Discussion

This case describes an exceptionally uncommon initial presentation of HL characterized by predominant hepatic involvement and a cholestatic clinical picture that closely mimicked a range of hepatobiliary, infectious, and inflammatory conditions. The diagnosis was ultimately established through liver core biopsy, which is an uncommon but critical diagnostic modality for HL in this context. In this case, the patient presented with progressively worsening jaundice as a primary complaint. Jaundice is a rare initial presentation of HL, occurring in only about 1-2% of cases, and is most often associated with hepatic involvement or paraneoplastic syndromes such as vanishing bile duct syndrome. The rarity is supported by retrospective studies showing that only 1.4% of newly diagnosed HL patients presented with cholestatic febrile illness and that jaundice was present in just 6.1% of cases with histologically confirmed liver involvement, while symptoms of lymphadenopathy, fever, and weight loss presented more frequently [5,7]. Liver biopsy plays a crucial role in establishing the diagnosis of HL in patients with unexplained cholestatic jaundice, especially when noninvasive modalities are inconclusive. The National Comprehensive Cancer Network (NCCN) guidelines recommend excisional lymph node biopsy as the standard but acknowledge that core needle or liver biopsy may be necessary when liver involvement is suspected and other sites are inaccessible [8]. Literature and guidelines emphasize that liver biopsy is particularly indicated when the etiology of jaundice remains unclear after laboratory and imaging workup, as it can confirm hepatic lymphoma or paraneoplastic syndromes such as vanishing bile duct syndrome [9]. The literature further confirms that core needle biopsy provides high diagnostic accuracy (typically above 90%) and is especially valuable for deep-seated or inaccessible lesions, including intra-abdominal or hepatic sites [10]. When liver involvement is suspected and other sites are inaccessible, liver biopsy is appropriate for diagnosis. In our case, the clinical presentation posed diagnostic challenges. The patient presented with worsening jaundice associated with fever and weight loss, with elevated cholestatic enzymes, required multiple diagnostic modalities, and was ultimately diagnosed by liver biopsy. About 1-2% of cases of HL are identified by liver biopsy, particularly when liver involvement is suspected and liver abnormalities appear on presentation. In a retrospective study, only six (1.4%) of the 421 HL patients were diagnosed by liver biopsy, as they presented with liver-related symptoms such as cholestasis or jaundice [5], highlighting the essential role of liver biopsy in establishing the diagnosis of HL. This case highlights the importance of considering HL in patients who present with jaundice as a primary complaint, especially in the setting of inconclusive laboratory and imaging tests. The rarity of atypical presentations requires a high index of suspicion to avoid delayed diagnosis. Maintaining a high index of suspicion for lymphoma in patients with unexplained cholestatic jaundice, and utilizing liver biopsy when indicated, is essential for timely diagnosis and management.

## Conclusions

This case underscores a rare initial presentation of classical HL manifesting as cholestatic jaundice due to hepatic involvement. Such presentations often mimic hepatobiliary, infectious, or autoimmune disorders, leading to diagnostic delay. When initial laboratory and imaging studies fail to identify the etiology of liver dysfunction, clinicians should maintain a high index of suspicion for infiltrative malignancies.

Liver biopsy remains a pivotal diagnostic tool in such scenarios, providing definitive histopathologic confirmation when other diagnostic modalities are inconclusive. Early recognition and tissue diagnosis are essential for prompt initiation of therapy and improved clinical outcomes. This case reinforces the importance of considering HL in the differential diagnosis of unexplained cholestatic jaundice.
